# Diagnostic Efficacy of PET/CT-Aided *versus* Conventional CT-guided Lung Biopsy: A Systematic Review and Meta-Analysis

**DOI:** 10.2174/0115734056394487250702094607

**Published:** 2025-07-15

**Authors:** Yeonhee Lee, Sowon Jang, Minseon Kim, Junghoon Kim

**Affiliations:** 1 Department of Internal Medicine, Seoul National University Hospital, Seoul, South Korea; 2 Department of Radiology, Seoul National University Bundang Hospital, Seongnam, South Korea

**Keywords:** Biopsy, Lung biopsy, CT, PET/CT, Diagnostic Efficacy, Meta-analysis, Risk ratios

## Abstract

**Introduction:**

Unlike its well-established role in lung cancer staging, positron emission tomography /computed tomography (PET/CT)'s role in guiding lung biopsies remains unclear and underutilized, despite its potential to distinguish metabolically active regions from areas of necrosis or fibrosis within lesions.

**Objective:**

This study aims to assess the diagnostic efficacy of PET/CT-aided *versus* conventional CT-guided lung biopsy by comparing the incidences of non-diagnostic results, false results, and complications.

**Methods:**

Studies comparing PET/CT-aided and conventional CT-guided lung biopsy were identified through an intensive search of PubMed, Embase, and the Cochrane Library. Data on nondiagnostic results, false results, and complications were extracted. Risk ratios (RRs) with 95% confidence intervals (CIs) were calculated using a random-effects model.

**Results:**

Seven studies involving 1,661 procedures were included. PET/CT-aided lung biopsy significantly reduced nondiagnostic results compared to conventional CT-guided biopsy (2.8% *vs*. 9.1%; pooled RR: 0.38, 95% CI: 0.20–0.70, P = 0.002). False results were also significantly fewer in the PET/CT-aided group (6.5% *vs*. 17.0%; pooled RR: 0.48, 95% CI: 0.35–0.65, P < 0.001). There was no statistically significant difference in overall complication rates (28.1% *vs*. 32.5%; pooled RR: 0.92, 95% CI: 0.77–1.10, P = 0.352), while PET/CT-aided biopsy showed a slight tendency toward fewer major complications (0.9% *vs*. 1.7%; pooled RR: 0.67, 95% CI: 0.30–1.44, P = 0.303).

**Conclusion:**

PET/CT-aided CT-guided lung biopsy offers advantages over conventional CT-guided lung biopsy by significantly reducing nondiagnostic and false results, without significant differences in the risk of complications.

## 1 INTRODUCTION

Image-guided lung biopsy is widely recognized as a reliable and safe technique for obtaining specimens from lung lesions, especially when malignancy is suspected [1-4]. Traditionally, image-guided lung biopsies have been performed using anatomical imaging modalities such as computed tomography (CT), fluoroscopy, CT fluoroscopic imaging, and ultrasound to enhance the procedural precision. The primary goal of image-guided lung biopsy is to obtain a tissue sample of adequate quality, ensuring a safe and accurate diagnosis that ideally meets or exceeds established diagnostic accuracy standards [5, 6]. The accuracy of lesion sampling is of paramount importance not only in diagnosis but also in assessing prognosis and personalizing treatment plans, especially in the era of personalized medicine, which includes tumor genotyping and targeted therapies [7-9]. However, traditional image-guided biopsies rely solely on anatomical imaging, limiting their ability to differentiate between viable tumors and areas of inflammation or necrosis. This limitation can lead to nondiagnostic or inconclusive biopsy outcomes [10, 11].

Owing to its capability of revealing the metabolic activity patterns within lesions, positron emission tomography/computed tomography (PET/CT) has become a critical tool in lung cancer diagnosis, staging, and management [12-16]. By combining anatomical and metabolic imaging, PET/CT enhances the detection of malignant lesions, improves tumor characterization, and provides high diagnostic accuracy in lung cancer staging. Current guidelines recommend PET/CT for lung cancer staging and therapy planning due to its ability to identify metabolically active regions of tumors and detect distant metastases [17-24].

Unlike its well-established role in lung cancer staging, PET/CT's role in guiding lung biopsies remains unclear and underutilized in current clinical practice, despite its potential to distinguish metabolically active regions from areas of necrosis or fibrosis within lesions [25-27]. Several studies have compared the diagnostic performance of PET/CT-aided and conventional CT-guided lung biopsies [25, 28-39]. These studies suggest that PET/CT guidance may improve diagnostic accuracy by targeting metabolically active regions within lesions while reducing the risk of sampling errors. However, no extensive cohort studies or meta-analyses have been conducted on this topic. Therefore, we conducted a meta-analysis of studies comparing PET/CT-aided and conventional CT-guided lung biopsies to directly compare the diagnostic efficacy of the two methods.

## 2 MATERIALS AND METHODS

This systematic review adhered to the Preferred Reporting Items for Systematic Reviews and Meta-Analyses of Diagnostic Test Accuracy Studies (PRISMA-DTA). Approval from the institutional review board and informed consent were not required for this study, as it is a systematic review and meta-analysis of published data. The data used are available from the authors upon request.

### 2.1 Literature Search

An intensive systematic search was performed in PubMed, Embase, and the Cochrane Library databases to identify studies comparing the diagnostic performance of PET/CT-aided and conventional CT-guided biopsy for identifying lung lesions, particularly malignant neoplasms. The literature search was conducted using the following keywords in titles and abstracts: (OR 'lung' OR 'transthoracic' OR 'pulmonary') AND ('biops*' OR 'aspiration') AND 'computed tomography' AND 'positron emission tomography'. These terms were combined using appropriate Boolean operators to optimize the search results. The latest literature search was conducted on 31 December 2024.

### 2.2 Study Selection

Two authors (J.K., YL) independently assessed titles and abstracts for eligibility. In cases of disagreement over study eligibility, consensus was achieved through discussion with another co-author (S.J.).

#### 2.2.1 Inclusion Criteria

Studies were considered if they evaluated the diagnostic performance of PET/CT-aided lung biopsy in comparison to conventional CT-guided lung biopsy, with a focus on distinguishing malignant lesions. This selection criterion was implemented to reduce potential biases both within and between studies. After a full-text review, studies were included based on their provision of adequate data to calculate the number of nondiagnostic or false results. A nondiagnostic result was defined as a pathologically inconclusive biopsy due to technical failure or an inadequate specimen. A false result was defined as a mismatch between the biopsy result and the final diagnosis regarding malignancy.

#### 2.2.2 Exclusion Criteria

Non-English studies, case reports or series, conference abstracts, or reviews were excluded. Studies evaluating only single-index tests and those not providing distinct data sets for PET/CT-aided and conventional CT-guided lung biopsies were excluded from consideration.

### 2.3 Quality Assessment of the Included Studies

The methodological quality of the selected studies was assessed independently by the two authors (J.K., Y.L.) using Quality Assessment of Diagnostic Accuracy Studies (QUADAS-2) criteria to evaluate the risk of bias and the applicability of the included studies. In case of discrepancies, consensus was achieved through discussion with another co-author (M.K.).

### 2.4 Data Extraction

The total number of biopsies, as well as the numbers of nondiagnostic results, false results, and overall and major complications for both PET/CT-aided and conventional CT-guided lung biopsies, were extracted. In addition, the following data were extracted for each study: characteristics of the included studies, patient gender and age, biopsy needle size, biopsy performer, PET/CT application method, interval between PET/CT and biopsy, lesion size, and proportion of malignant lesions in the final diagnosis. Data extraction from the studies was independently performed by two authors (J.K., Y.L.) using a standardized form. In cases of disagreement, consensus was achieved through discussion with another investigator (S.J.).

### 2.5 Statistical Analysis

Statistical analyses were conducted using R version 4.3.2 (R Foundation for Statistical Computing) and STATA version 16 (StataCorp). A random-effects model was employed to meta-analyze risk ratios (RRs) for nondiagnostic results, false-positive results, and complications, along with 95% confidence intervals (CIs). Heterogeneity among studies was quantified through Higgins' I^2^ statistic, where values of 25–49%, 50–74%, and ≥ 75% were interpreted as low, moderate, and high heterogeneity, respectively. To investigate potential sources of heterogeneity and to determine whether specific factors influence the effectiveness of the biopsy, subgroup analyses were performed. Publication bias was evaluated through Egger's test to detect asymmetry in the funnel plot. Statistical significance was defined as a P-value of less than 0.05 for all analyses.

## 3 RESULTS

### 3.1 Study Selection

**Fig. (1):**
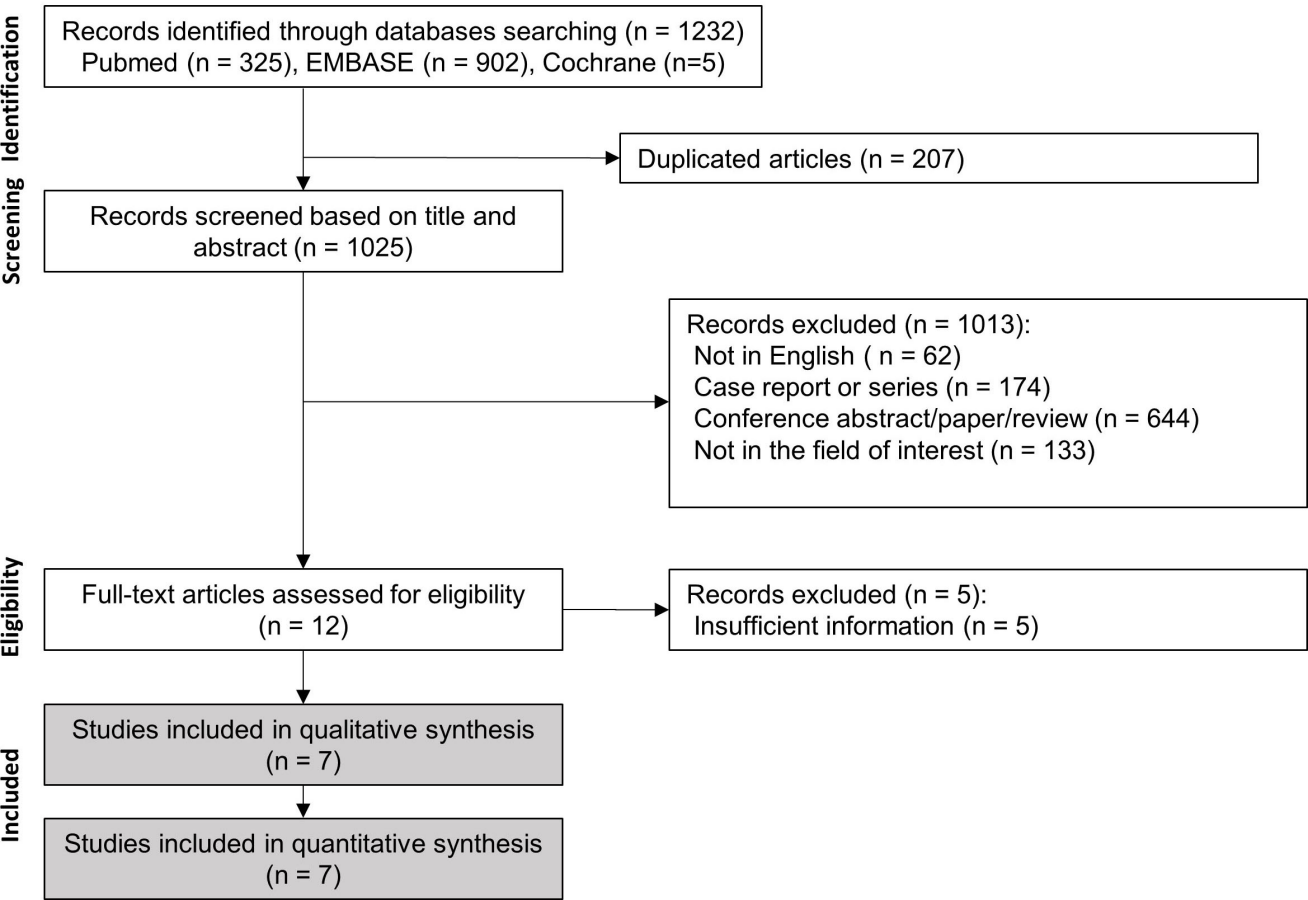
Study search and selection.

Our search of the online literature yielded 1,232 studies: 325 in PubMed, 902 in Embase, and 5 in the Cochrane Library. After removing duplicates, 1,025 studies remained. Upon reviewing the titles and abstracts of the 1,025 studies, 1,013 were removed based on the following criteria: non-English studies (n = 62); case reports or series (n = 174); conference abstracts, papers, or reviews (n = 644); and not in the field of interest (n = 133). After a full-text review, five studies were excluded due to insufficient data [28-32]. Finally, seven studies that collectively reported on 1,661 procedures were included (Fig. **1**). The characteristics of the selected studies are presented in Table **1**.

### 3.2 Quality Assessment of the Included Studies

Table **2** presents a summary of the risk of bias and applicability concerns for all the included studies, according to the QUADAS-2 criteria. In the patient selection domain, a high risk of bias was assigned to case-control studies. The index test domain was rated as having high applicability concerns if the biopsy used CT-PET/CT fusion image guidance, due to lower general applicability. A clinical follow-up period shorter than six months for patients with negative biopsy results was considered a high risk of bias in the reference standard domain. Studies lacking clear documentation of the relationship between PET/CT imaging and CT-guided lung biopsy were classified as having an unknown risk of bias in the flow and timing domain.

### 3.3 Nondiagnostic Results of PET/CT-aided *versus* Conventional CT-guided Lung Biopsy

The overall pooled incidence of nondiagnostic results across both lung biopsy techniques was 5.8% (95% CI: 2.6%–10.1%). The pooled incidence of nondiagnostic results for PET/CT-aided CT-guided lung biopsy was 2.8% (95% CI: 0.1%–8.0%), whereas that for conventional CT-guided lung biopsy was 9.1% (95% CI: 6.1%–12.3%). Compared to conventional CT-guided lung biopsy, PET/CT-aided CT-guided lung biopsy was associated with significantly fewer nondiagnostic results, with a pooled RR of 0.38 (95% CI: 0.20–0.70, *P* < 0.001). No significant heterogeneity was detected (I^2^ = 11.0%, *X*^2^ P = 0.343). Fig. (**2**) presents the forest plot of the RRs for nondiagnostic results, along with the total number of cases and the corresponding number of nondiagnostic results in each study.

### 3.4 False Results of PET/CT-aided *versus* Conventional CT-guided Lung Biopsy

The overall pooled incidence of false results for both PET/CT-aided and conventional CT-guided lung biopsy was estimated at 11.8% (95% CI: 6.7%–18.1%). The pooled incidences of false results for PET/CT-aided and conventional CT-guided lung biopsy were 6.5% (95% CI: 1.8%–13.4%) and 17.0% (95% CI: 11.8%–22.9%), respectively. In comparison to conventional CT-guided lung biopsy, PET/CT-aided CT-guided lung biopsy was associated with significantly fewer false results, with a pooled RR of 0.48 (95% CI: 0.35–0.65, *P* < 0.001). No significant heterogeneity was observed (I^2^ = 0%, *X*2 *P* = 0.416). Fig. (**3**) presents the forest plot of RRs for false results, as well as the total number of cases and false results reported in each study.

### 3.5 Subgroup Analysis

Subgroup analyses were performed based on PET application methods (pre-procedural and fusion), needle size (18G and 20G), and lesion size (≥ 4 cm and < 4 cm). The subgroup analysis of nondiagnostic results showed that pre-procedural PET/CT guidance significantly improved diagnostic outcomes by reducing the risk compared to conventional methods (P = 0.008). At the same time, fusion PET application did not present statistically significant results (P = 0.334). However, the test of group differences revealed no statistically significant difference between fusion and pre-procedural PET/CT application methods (P = 0.701). While there were trends toward reduced nondiagnostic results for both smaller and larger lesions and both needle sizes, these were not statistically significant. In the test of group differences, there was no statistically significant difference based on needle size (P = 0.324) or lesion size (P = 0.273). In the subgroup analysis for false results, pre-procedural PET application significantly reduced false results (P < 0.001), whereas fusion PET application did not show a significant effect (P = 0.178) when compared to conventional CT-guided biopsy. However, the intergroup difference between pre-procedural and fusion PET applications was not significant (P = 0.993). Both needle sizes and both smaller and larger lesions showed significant reductions in false results, without significant intergroup differences (P = 0.836 for needle size, P = 0.479 for lesion size). The results of the subgroup analyses are summarized in Table **3**.

### 3.6 Heterogeneity Analysis

The results for PET/CT-aided and conventional CT-guided lung biopsy showed no significant heterogeneity (I^2^ ≤ 11%). Publication bias was evaluated using Egger's test for funnel plot asymmetry. The test revealed a biased estimate of -1.00 (SE = 1.24), with a t-value of -0.81 (df = 3). The resulting p-value of 0.478 was not statistically significant, suggesting no strong evidence of publication bias in our meta-analysis. The funnel plot illustrating these results is presented in Fig. (**4**).

### 3.7 Post-procedure Complications

The overall complication rate for lung biopsies, combining both PET/CT-aided and conventional CT-guided techniques, was found to be 30.3% (95% CI: 8.2%–58.8%). The pooled incidence of overall complications for PET/CT-aided CT-guided lung biopsy was 28.1% (95% CI: 6.3%–57.6%), while the incidence for conventional CT-guided lung biopsy was 32.5% (95% CI: 10.1%–60.1%). No significant difference in overall complication risk was found between PET/CT-aided and conventional CT-guided lung biopsy, with a pooled RR of 0.92 (95% CI: 0.77–1.10, *P* = 0.352). Significant heterogeneity was not observed (I^2^ = 0.0%, *X*2 *P* = 0.455). In terms of major complications, the pooled incidence for both PET/CT-aided and conventional CT-guided lung biopsy was estimated at 1.4% (95% CI: 0.0%–5.2%). The pooled incidence of major complications of PET/CT-aided and conventional CT-guided lung biopsies was 0.9% (95% CI: 0.0%–4.2%) and 1.7% (95% CI: 0.0%–6.4%), respectively. In comparison to conventional CT-guided lung biopsy, PET/CT-aided CT-guided lung biopsy was associated with a slight tendency toward fewer major complications, with a pooled RR of 0.67 (95% CI: 0.30–1.44, *P* = 0.303). No significant heterogeneity was observed (I^2^ = 0%, *X*2 *P* = 0.881). The forest plots of the RRs for overall and major complications are presented in Figs. (**5** and **6**), respectively.

## 4 DISCUSSION

**Fig. (2):**
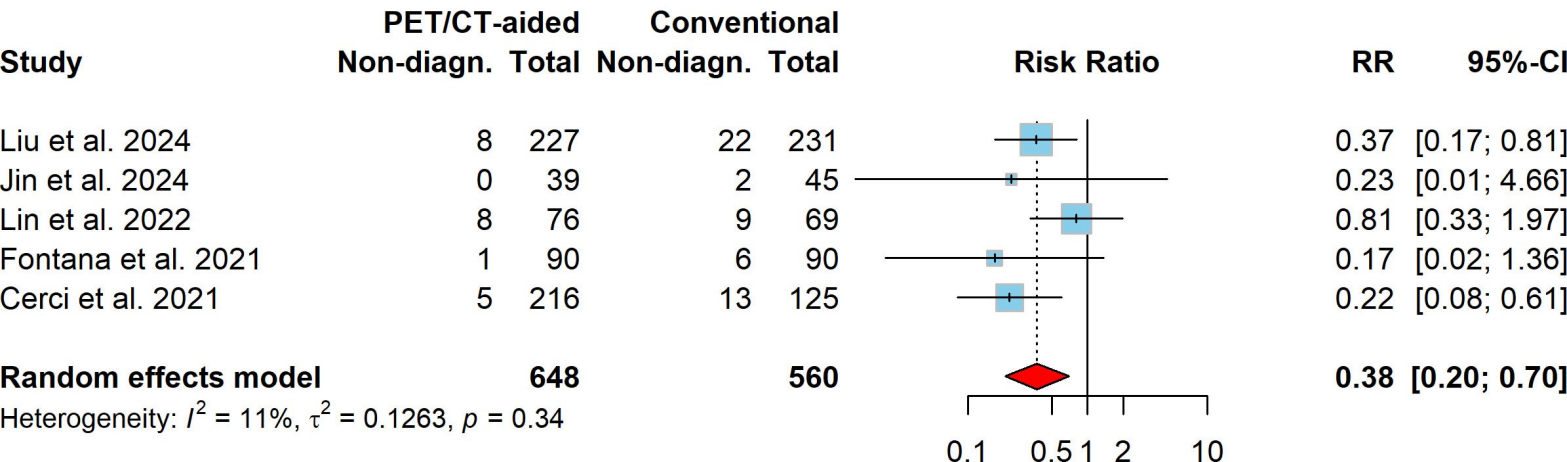
Forest plot comparing the incidence of nondiagnostic results between PET/CT-aided and conventional CT-guided lung biopsies across the included studies. Squares and horizontal lines represent individual study risk ratios (RRs) and their 95% confidence intervals (CIs); the diamond indicates the pooled RR. Values <1 favor PET/CT-aided biopsy.

**Fig. (3):**
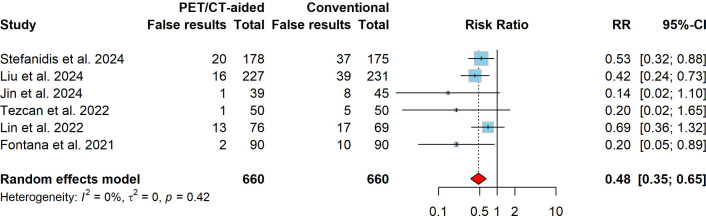
Forest plot comparing the incidence of false results between PET/CT-aided and conventional CT-guided lung biopsies across the included studies. Squares and horizontal lines represent individual study risk ratios (RRs) and their 95% confidence intervals (CIs); the diamond indicates the pooled RR. Values <1 favor PET/CT-aided biopsy.

This systematic review and meta-analysis evaluated the diagnostic outcomes and complications of PET/CT-aided CT-guided lung biopsies in comparison to conventional CT-guided lung biopsies for diagnosing pulmonary lesions, particularly malignancies. The pooled incidence of nondiagnostic results for both PET/CT-aided and conventional CT-guided lung biopsy was 5.8% (95% CI: 2.6%–10.1%), which aligns with a meta-analysis that reported a pooled nondiagnostic rate of 6.8% (95% CI: 6.0%–7.6%) for percutaneous transthoracic needle biopsies [40]. The pooled analysis demonstrated that PET/CT-aided CT-guided lung biopsy significantly reduced the incidence of nondiagnostic results compared to conventional CT-guided biopsy (2.8% *vs*. 9.1%; pooled RR: 0.38, 95% CI: 0.20–0.70, P = 0.002; (Fig. **2**), supporting the role of PET/CT in improving diagnostic yield by avoiding necrotic or non-representative areas during biopsy [25, 27, 29, 35, 36, 39]. Additionally, the pooled incidence of false results was significantly lower in the PET/CT-aided group (6.5% *vs*. 17.0%; pooled RR: 0.48, 95% CI: 0.35–0.65, P < 0.001; Fig. **3**). This finding aligns with previous studies suggesting that PET/CT imaging enhances the ability to target metabolically active regions within lesions, thereby improving diagnostic accuracy and reducing false-negative rates [31, 35, 36, 41].

In the subgroup analysis, pre-procedural PET application significantly reduced false results (P < 0.001) and improved diagnostic outcomes for nondiagnostic results (P = 0.008), demonstrating its effectiveness at enhancing biopsy accuracy. In contrast, fusion PET application did not show statistically significant results for either false results (P = 0.178) or nondiagnostic results (P = 0.334). However, the intergroup difference between pre-procedural and fusion PET applications was not significant for false results (P = 0.993) or nondiagnostic results (P = 0.701). The lack of significance in reducing nondiagnostic and false results for fusion PET application may be attributed to smaller sample sizes and the variability across studies in this subgroup, which could limit the statistical power to detect meaningful effects. The test of intergroup differences between 18G and 20G needles, as well as between smaller (< 4 cm) and larger (≥ 4 cm) lesions, did not reveal significant differences (Table **3**).

**Fig. (5):**
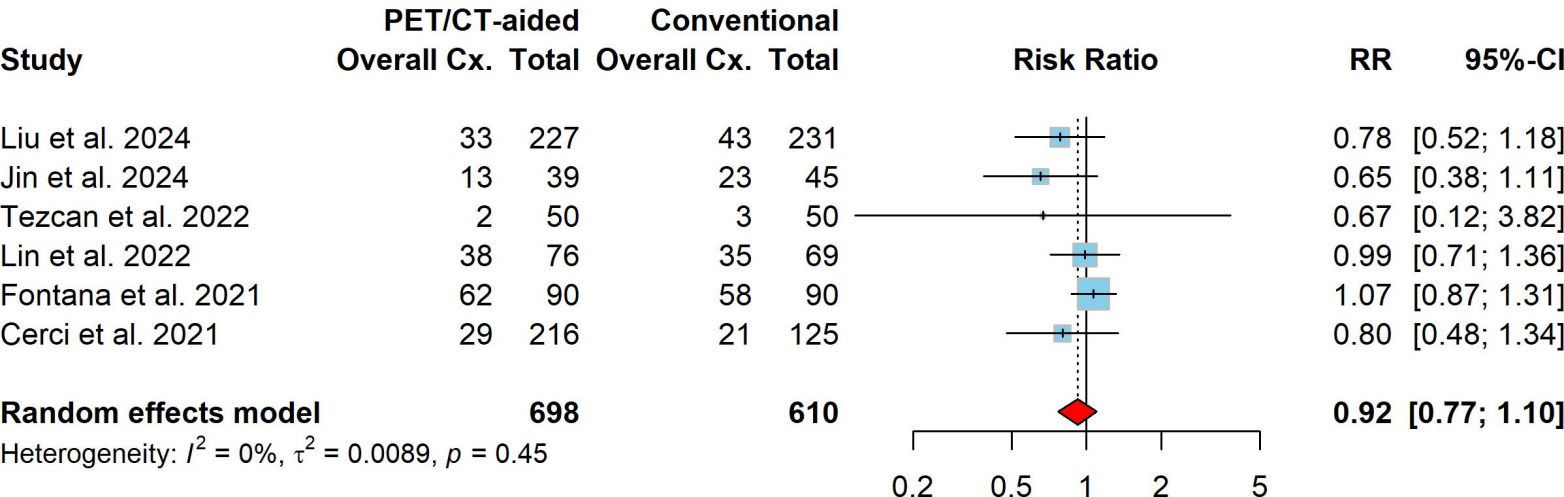
Forest plot comparing overall complication rates between PET/CT-aided and conventional CT-guided lung biopsies. Squares and horizontal lines represent individual study risk ratios (RRs) and their 95% confidence intervals (CIs); the diamond indicates the pooled RR.

**Fig. (6):**
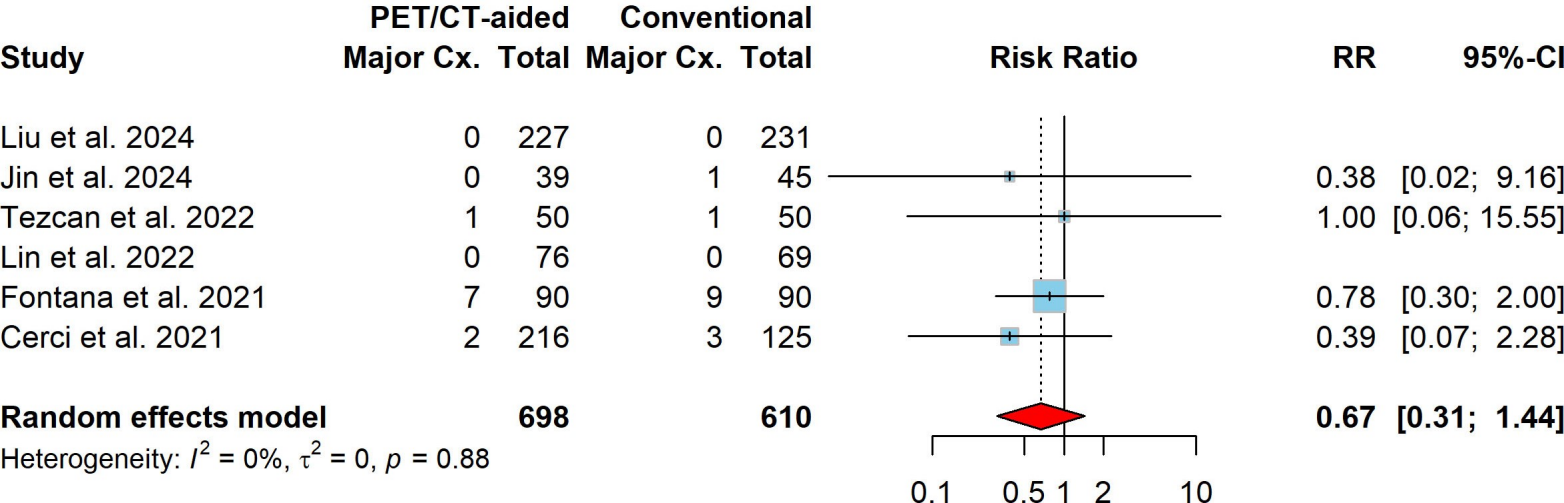
Forest plot comparing major complication rates between PET/CT-aided and conventional CT-guided lung biopsies. Squares and horizontal lines represent individual study risk ratios (RRs) and their 95% confidence intervals (CIs); the diamond indicates the pooled RR.

In terms of complications, the pooled incidence of overall complications for both PET/CT-aided and conventional CT-guided lung biopsy was estimated at 30.3% (95% CI: 8.2%–58.8%), which is comparable to the overall complication rate reported in a meta-analysis for percutaneous lung biopsy (43%, 95% CI: 25%–62%) [4]. No significant difference in overall complication risk was found between PET/CT-aided and conventional CT-guided lung biopsy (28.1% *vs*. 32.5%; pooled RR: 0.92, 95% CI: 0.77–1.10, P = 0.352; Fig. **5**). However, the pooled incidence of major complications tended to be slightly lower in the PET/CT-aided group than in the conventional group (0.9% *vs*. 1.7%; pooled RR: 0.67, 95% CI: 0.31–1.44, P = 0.303; Fig. **6**). These findings align with studies indicating that PET/CT guidance may reduce complications by minimizing unnecessary needle passes and by targeting safer biopsy sites [27, 29, 34, 37, 42]. The analyses revealed low heterogeneity across studies for all assessed outcomes (I^2^ ≤ 11%), indicating consistency in the findings across the included studies. Furthermore, Egger's test for funnel plot asymmetry revealed no strong publication bias (bias estimate: -1.00, SE = 1.24, t = -0.81, P = 0.478), suggesting that the results are unlikely to have been influenced by selective reporting.

The results highlight the clinical utility of incorporating prior PET/CT imaging into CT-guided lung biopsy workflows when feasible, particularly in cases that require precise lesion targeting for malignancy diagnosis. By enabling the precise targeting of metabolically active regions within lesions, PET/CT guidance offers a valuable tool for reducing nondiagnostic and false-positive results, which can minimize the need for repeat procedures and facilitate more informed clinical decision-making [29, 34, 43]. This is particularly relevant in cases involving complex or ambiguous lesions where conventional CT guidance alone may be insufficient for accurate diagnosis. Additionally, PET/CT imaging has been reported not only to improve diagnostic accuracy but also to reduce the procedure time of lung biopsies by optimizing needle placement [34, 36].

## STUDY LIMITATIONS

This study had several potential limitations. The primary limitation was the relatively limited number of included studies that provided direct comparisons between PET/CT-aided and conventional CT-guided lung biopsy within the same medical center. However, we anticipated that adopting this strict inclusion criterion, rather than comparing independent studies on PET/CT-aided and conventional CT-guided lung biopsies, could minimize intra- and inter-study biases and improve the validity of comparisons between the two biopsy approaches. Second, most of the included studies did not provide sufficient data to construct 2-by-2 contingency tables, precluding calculation of pooled sensitivity, specificity, and other diagnostic accuracy measures. This lack of detailed data limited the comprehensiveness of analyses and comparison of diagnostic accuracy between PET/CT-aided and conventional CT-guided lung biopsies. Third, variability in procedural techniques, patient populations, and follow-up durations across studies could have introduced unmeasured confounders. Lastly, this meta-analysis of diagnostic test accuracy encountered common challenges, including selection bias, publication bias, and missing data in certain studies. Although Egger's test for funnel plot asymmetry did not reveal significant publication bias, this does not entirely rule out its presence. The exclusion of non-English studies may have further increased the risk of publication bias.

## CONCLUSION

PET/CT-aided CT-guided lung biopsy offers advantages over conventional CT-guided lung biopsy by significantly reducing nondiagnostic and false results, with no significant differences observed in the risk of complications.

## AUTHOR'S CONTRIBUTIONS

The authors confirm their contribution to the paper as follows: J.K.: Study conception and design; S.J., M.K.: Analysis and interpretation of results; Y.L.: Draft manuscript. All authors reviewed the results and approved the final version of the manuscript.

## Figures and Tables

**Fig. (4):**
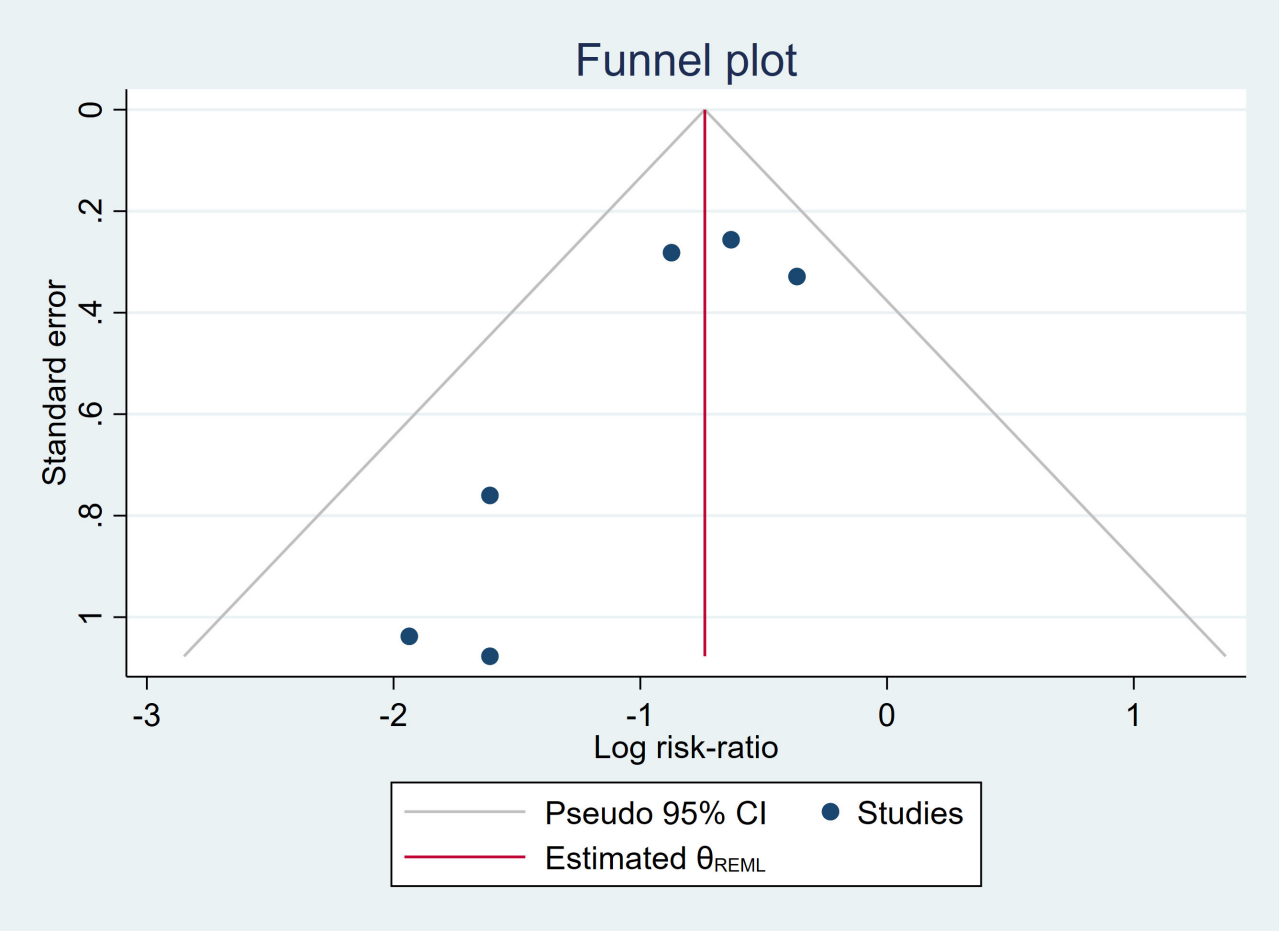
Funnel plot assessing publication bias for the included studies.

**Table 1:** Study characteristics.

**Study\Refs.**	**Year**	**Country**	**Study Design**	**Needle Size** **(Gauge)**	**Biopsy Performed by**	**PET Application**	**PET-Bx. interval (days)**	**Group**	**Sample Size** **(No.)**	**Mean Age** **(yrs)**	**Male Patients** **(%)**	Lesion Size * **(mm)**	**Malignant Lesion (%)**
Stefanidis *et al*.	2024	United Kingdom	Retrospective	20	Thoracic radiologists	Pre-procedural PET/CT	21	PET/CT-aided Conventional	178 175	70.9 80.0	N/A N/A	26.5 (18.0–46.0) 31.0 (20.0–47.0)	94.4 97.1
Liu *et al*.	2024	China	Retrospective	18	Nuclear medicine specialists	Pre-procedural PET/CT	7	PET/CT-aided Conventional	227 231	64.2 63.5	51.1 59.3	41.0 (26.5–55.5) 40.0 (26.0–54.0)	85.5 78.4
Jin *et al*.	2024	China	Retrospective	18	Physician	Pre-procedural PET/CT	15	PET/CT-aided Conventional	39 45	61.2 63.2	53.8 66.7	32.8 ± 14.0 33.4 ±13.9	82.1 77.8
Tezcan *et al*.	2022	Türkiye	Prospective	18	N/A	Pre-procedural PET/CT	N/A	PET/CT-aided Conventional	50 50	65.9 68.1	88.0 82.0	57.2 ± 27.9 52.9 ± 32.1	96.0 72.0
Lin *et al*.	2022	China	Retrospective	18	Interventional radiologists	CT-PET/CT fusion	14	PET/CT-aided Conventional	76 69	65.1 60.8	72.4 63.8	51.8 ± 21.9 50.8 ± 18.8	69.7 69.6
Fontana *et al*.	2021	Italy	Retrospective	20	Interventional radiologists	CT-PET/CT fusion	N/A	PET/CT-aided Conventional	90 90	70 72	68.9 71.1	35.0 (21.2–46.7) 26.0 (17.0–46.7)	81.1 85.6
Cerci *et al*.	2021	Brazil	Prospective	N/A	Physician	Intraprocedure PET/CT	0	PET/CT-aided Conventional	216 125	65.4 65.9	49.5 48.0	N/A N/A	81.0 71.2

**Abbreviations:** PET – positron emission tomography, Bx. – biopsy, N/A – not applicable. * Data are presented as provided in the studies: “median (interquartile range)” or “mean ± standard deviation”

**Table 2:** Assessment of reporting quality by QUADAS-2 scoring system.

Study	Risk of Bias							Applicability Concerns		
	Patient Selection	Index Test		Reference Standard		Flow and Timing		Patient Selection	Index Test	Reference Standard
Stefanidis *et al*. (2024)	ϑ	ϑ		ϑ		ϑ		ϑ	ϑ	ϑ
Liu *et al*. (2024)	ϑ	ϑ		ϑ		?		ϑ	ϑ	ϑ
Jin *et al*.(2024)	Λ	ϑ		ϑ		ϑ		ϑ	ϑ	ϑ
Tezcan *et al*. (2022)	Λ	ϑ		Λ		ϑ		ϑ	ϑ	ϑ
Lin *et al*. (2022)	ϑ	ϑ		ϑ		ϑ		ϑ	Λ	ϑ
Fontana *et al*. (2021)	ϑ	ϑ		ϑ		ϑ		ϑ	Λ	ϑ
Cerci *et al*. (2021)	ϑ	ϑ		ϑ		?		ϑ	Λ	ϑ

ϑLow Risk ΛHigh Risk ? Unclear Risk

**Table 3:** Subgroup analysis of log risk-ratio for false and nondiagnostic results.

-	No. of Studies	Log Risk-ratio [95% Confidence Interval]	P-value
Nondiagnostic results			
PET application methods			0.701
Pre-procedural	2	-1.025 [-1.787, -0.262]	0.008*
Fusion	2	-0.706 [-2.139, 0.726]	0.334
Needle size			0.324
18G	3	-0.681 [-1.377, 0.014]	0.055
20G	1	-1.792 [-3.889, 0.305]	0.094
Lesion size			0.273
≥ 4 cm	2	-0.634 [-1.396, 0.128]	0.094
< 4 cm	2	-1.686 [-3.406, 0.033]	0.055
False results			
PET application methods			0.993
Pre-procedural	4	-0.804 [-1.164, -0.444]	< 0.001*
Fusion	2	-0.798 [-1.961, 0.364]	0.178
Needle size			0.836
18G	4	-0.760 [-1.230, -0.290]	0.002*
20G	2	-0.859 [-1.667, -0.050]	0.037*
Lesion size			0.479
≥ 4 cm	3	-0.695 [-1.159, -0.231]	0.003*
< 4 cm	3	-1.044 [-1.891, -0.196]	0.016*

* P < 0.05. **Abbreviations:** PET – positron emission tomography.

## Data Availability

The data and supportive information are available within the article.

## LIST OF ABBREVIATIONS

CT: Computed Tomography
PET/CT: Positron Emission Tomography/Computed Tomography
RR: Risk Ratio
CI: Confidence Interval

## STANDARDS OF REPORTING

PRISMA guidelines and methodology were followed.
